# A procedure for Dex-induced gene transactivation in Arabidopsis ovules

**DOI:** 10.1186/s13007-022-00879-x

**Published:** 2022-03-29

**Authors:** Jasmin Schubert, Yanru Li, Marta A. Mendes, Danli Fei, Hugh Dickinson, Ian Moore, Célia Baroux

**Affiliations:** 1 Institute of Plant and Microbial Biology and Zürich-Basel Plant Science Center, University of Zürich, Zollikerstrasse 107, 8008 Zurich, Switzerland; 2 Dipartimento di Bioscienze, Universitá degli Studi di Milano, 20133 Milan, Italy; 3 Department of Plant Sciences, University of Oxford, Oxford, OX1 3RB UK

**Keywords:** pOP/LhGR, Dex induction, Ovule development, Arabidopsis

## Abstract

**Background:**

Elucidating the genetic and molecular control of plant reproduction often requires the deployment of functional approaches based on reverse or forward genetic screens. The loss-of-function of essential genes, however, may lead to plant lethality prior to reproductive development or to the formation of sterile structures before the organ-of-interest can be analyzed. In these cases, inducible approaches that enable a spatial and temporal control of the genetic perturbation are extremely valuable. Genetic induction in reproductive organs, such as the ovule, deeply embedded in the flower, is a delicate procedure that requires both optimization and validation.

**Results:**

Here we report on a streamlined procedure enabling reliable induction of gene expression in Arabidopsis ovule and anther tissues using the popular pOP/LhGR Dex-inducible system. We demonstrate its efficiency and reliability using fluorescent reporter proteins and histochemical detection of the GUS reporter gene.

**Conclusion:**

The pOP/LhGR system allows for a rapid, efficient, and reliable induction of transgenes in developing ovules without compromising developmental progression. This approach opens new possibilities for the functional analysis of candidate regulators in sporogenesis and gametogenesis, which is otherwise affected by early lethality in conventional, stable mutants.

**Supplementary Information:**

The online version contains supplementary material available at 10.1186/s13007-022-00879-x.

## Background

To elucidate the genetic and molecular mechanisms of a developmental process, a conventional approach consists of analyzing mutant lines isolated in a forward- or reverse genetic screen. However, the stable expression of a gain- or a loss-of-function mutation in a gene acting at several stages of plant development can be deleterious prior to the formation of a tissue/organ of interest, thus impairing its analysis. This is a difficulty particularly faced when analyzing the role of essential genes with pleiotropic effects during reproduction. Their disruption following chemically-induced mutagenesis, insertional mutagenesis or gene editing may abort development or organ growth before reproductive development. In such cases, inducible transactivation systems offer an elegant solution for controlling a genetic perturbation in a conditional manner, be it an artificial miRNA for downregulation or an engineered mutant variant of the protein of interest. Combining a tissue-specific promoter driving the activating factor with the selective application of the inducer, such systems can provide exquisite temporal and spatial control. However, inducible systems often require that, both the expression control and application procedure must be adapted to the organ/tissue of interest and validated for functional analyses.

Different chemically-inducible system exist that rely on diverse compounds such as ethanol [1] or animal hormones, including estradiol [2] and dexamethasone [3]. Among them, the pOP/LhGR dexamethasone (Dex)-inducible system has been very popular and widely used. It relies on the Dex-induced translocation of the synthetic transcription factor LhGR into the nucleus. Prior to induction, LhGR is retained in the cytoplasm by the HSP90 chaperone but once in the nucleus, it binds to the pOP6 promoter at optimally-spaced repeats in [3]. The pOP/LhGR-inducible system had been demonstrated to induce tight and reliable gene expression control and has been widely used in Arabidopsis, Cardamine, tobacco and rice [4–12] and references therein. Reports of the successful use of the system include the induction of transcription factors, toxins for cell ablations, the cre/lox system for recombination, RNAi for gene downregulation and other cellular components, in leaves, roots, cambium tissue, zygote, embryo and apical meristems (see for instance [9, 10, 13–19] *inter alia*)*.* Resources have also been recently established of driver/responder lines for expression in different cell types in Arabidopsis and other species [8, 9]. Dex can be applied by watering whole plants, by foliar spray or supplementation in the growth medium of cell cultures, seedlings or tissue segments in vitro, [4, 9, 20]. However, localized induction in tissues that are embedded in floral organs poses technical difficulties due to their relative inaccessibility. Here we report on an application procedure that has been optimized for inducing transgene expression in developing ovules of *Arabidopsis*. This strategy largely preserves viability and ovule developmental progression. The solvent used for the Dex solution however induces a mild reduction in fertility, requiring the use of stringent mock controls. This application method allows for a durable (over several days) and efficient (throughout cell layers) transgene expression in developing ovules and is also applicable to anthers.

## Results

### Dexamethasone application on inflorescences

Ovule primordia are the site of the somatic-to-reproductive transition where in the female meiocyte differentiates [21]. In Arabidopsis, ovule primordia are small, digit-shape structures of 10–0 µM length depending on the developmental stage, encapsulated in the carpel itself, and enclosed in a flower bud [22]. Flower buds containing developing primordia are ca. 1–2 mm large and tightly packed in the apical inflorescence. Due to the relative inaccessibility of ovule primordia, chemical induction of gene expression is thus not trivial. Notably, the inducer must be applied locally on buds which can be brittle, and handling should ideally avoid wounding while enabling efficient penetration through the floral corolla (sepals, petals) and the carpel wall.

**Fig. 1:**
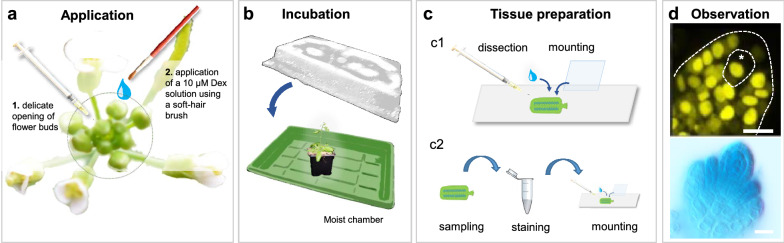
Schematic representation of steps involved to induce ovules of *Arabidopsis thaliana* with Dex. **a** Single floral buds are gently opened with dissecting needles under the stereomicroscope to allow for a good exposure of the carpel to the induction solution applied with a paint brush with soft hairs. **b** Plants are kept overnight in a covered tray, laying side-way, to prevent drying. Carpels are sampled at the desired time point following induction for direct microscopy analysis (**c1**) or for histochemical detection of the GUS reporter serving as induction control before downstream analyses [6] (**c2**). **D** Representative examples of induced reporter genes 16hpi: *RPS5a* >  > *nls3xmVenus* (top) and *RPS5a* >  > *GUS* (bottom)

We found that local application using a small soft-hair painting brush was not disruptive and allowed a precise application. Diluting Dex in a physiological medium such as half-MS was not sufficient to induce primordia inside the carpel. We found, however, that adding a surfactant such as Silwet^®^L-77 already used in Agrobacterium infiltration [23] enabled a good induction. Yet, excess of this surfactant led to necrosis and prevent floral bud development, as already reported [23]. Details of the procedure are as follows: petals and sepals of individual flower buds at the appropriate stage are gently pulled apart under a stereomicroscope using a dissecting needle, without damaging the bud, (Fig. 1a). Then, a drop of freshly prepared 10 µM Dex solution (or mock solution for control) is applied on the bud with a soft-hair paint brush. The application is monitored under the stereomicroscope and care is taken that the inflorescence remains wet but not engulfed in a large drop of induction medium (which would lead to tissue necrosis [23] overnight). After application, plants should be covered overnight (Fig. 1b). Keeping a moist environment prevents drying of the inducing medium before penetration into the tissue. In addition, because Dex is photosensitive [24], induction is preferably done in the evening. Manual application is delicate and variability between users may be observed in induction efficiency, particularly during the learning phase. This can be compensated by a second application in the morning of the next day (Additional file 2: Fig. S1).

Floral buds are then collected at the time-point of interest for downstream histological or molecular analyses (Fig. 1c, d).

We found that several factors influence the success of the induction: (i) *handling:* delicate handling is required for applying the solution and tissue injury may lead to flower bud arrest, visible in the next day by a paler color and loss of turgescence or on long term by sterility (see also later); we observed a user-dependent efficiency affecting the number of flower buds showing induction, probably during the application of the solution, a limitation which could be compensated by a second induction the next day (Additional file 2: Fig. S1); (ii) *age of the Dex solution*: the solution should be prepared freshly and stored at 4 °C for a maximum of three days. 10 mM Dex stock solutions either in DMSO or EtOH can be stored at – 20 °C. (iii) *plant health*: well-watered, healthy plants are better responding to the treatment, possibly corresponding to a better ability of the solution to penetrate in fresh, turgescent tissues, linked to this, well-developed adult plants grown several weeks in (small) pots and showing a weaker stature and possibly paler tissues provided less reliable induction (not shown); the primary inflorescence soon after emergence from the rosette is thus preferred for a reliable induction. (iv) *exposure to strong light*: Plants were grown with a light intensity of ~ 100 µE (μmol m^−2^ s^−1^) and the inflorescences were > 30 cm away from the illumination source; although not tested here, a stronger illumination could possibly reduce induction efficiency as Dex is photosensitive [24].

### Efficient induction in carpels and anthers of various reporter genes

**Fig. 2:**
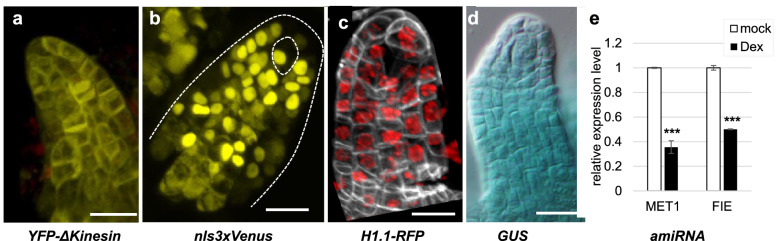
Induction of a variety of constructs with distinct subcellular targets. (**a**) *RPS5a* >  > *YFP-Δkinesin*; **b RPS5a** >  > *nls3xmVenus*. **c** *RPS5a* >  > *H1.1-RFP* (**d**) *RPS5a* >  > *GUS*. Ovule primordia (**a**–**d**) were dissected from flowers stage 9, sampled 24 hpi (**a**, **d**) or 5 dpi (**b**, **c**). **e** RT-PCR showing *FIE* and *MET1* expression levels in whole flower buds stage 6–10 (pooled) [43] expressing the inducible *SPL* >  > *amiRFIE or SPL* >  > *amiRMET1,* respectively. ***: 2-tailed t-test, P < 0.001. See Additional file 1 for raw and processed data. Scale bar = 10 µm

Under our conditions different reporters targeting distinct cellular compartments could be induced in the ovule primordium. Seventeen hours following the application of the inducer (17 hpi, hours post induction) we were able to detect robust expression of fluorescent proteins targeted in the endoplasmic reticulum (ER), (Fig. 2a), nucleoplasm (Fig. 2b), chromatin (Fig. 2c), or of the GUS reporter enzyme in the cytoplasm (Fig. 2d). To further validate these conditions functionally, we induced transgenes expressing amiRNA constructs targeting *FERTILIZATION INDEPENDENT ENDOSPERM1 (FIE)* and *METHYLTRANSFERASE1* (*MET1)* and confirmed a significant reduction in transcript levels (*p* < 0.001) in flower buds’ stage 6–10 (pooled) 48 h post induction (Fig. 2e).

**Fig. 3:**
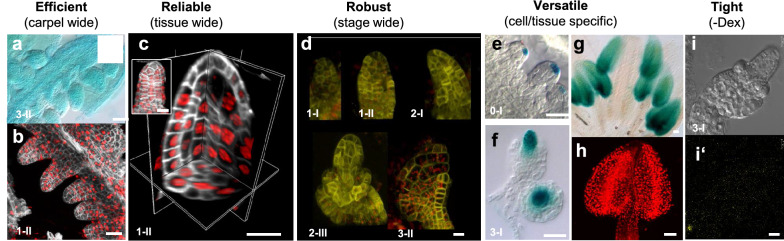
Induction in ovules and anthers. **a**–**h** Dex induction of the following lines: (**a**) *RPS5a* >  > *GUS,* (**b**, **c**, **h**) *RPS5a* >  > *H1.1-RFP* (**d**) *RPS5a* >  > *YFP-Δkinesin*, (**e**–**g**) *SPL* >  > *GUS.* (**i**–**i’**) Mock treatment of an *RPS5a* >  > *YFP-Δkinesin* line, in ovule primordia (**a**–**f**, **i**, stages are indicated according to the nomenclature [22]) or anthers from flower stage 12 (**g**, **h**). Time post induction: 24 h (**a**), 16 h (**b**–**i**). Confocal images: partial projections (**b**, **d**), full projection (c-inset, **h**), combined orthogonal sections (**c**); scale bar: 20 μm for all except **c**, 10 μm

For meaningful use in gene expression control in reproductive organs the chemical inducer must reach all tissue and cell types throughout the organ. The application protocol described here allows efficient induction of ovules throughout the carpel (Fig. 3a,b), throughout the cell layers of ovule primordia (Fig. 3c), was effective at different stages (Fig. 3d), following the expected cell-type specific expression (Fig. 3e) while being tightly regulated (Fig. 3h) as formerly described [3, 9]. When all conditions described earlier are met, 85–100% buds are induced and in those, all ovules show reporter signals (Additional file 2: Fig. S1).

### Effect of chemical induction on ovule development and plant fertility

One question arising from this application procedure is whether the mechanical and chemical treatment itself affects reproductive development. We therefore monitored three aspects: (i) the developmental progression of ovules in the carpel, (ii) the differentiation of the spore mother cell (SMC) in the ovule as a marker of functionality, and (iii) fertility.

**Fig. 4:**
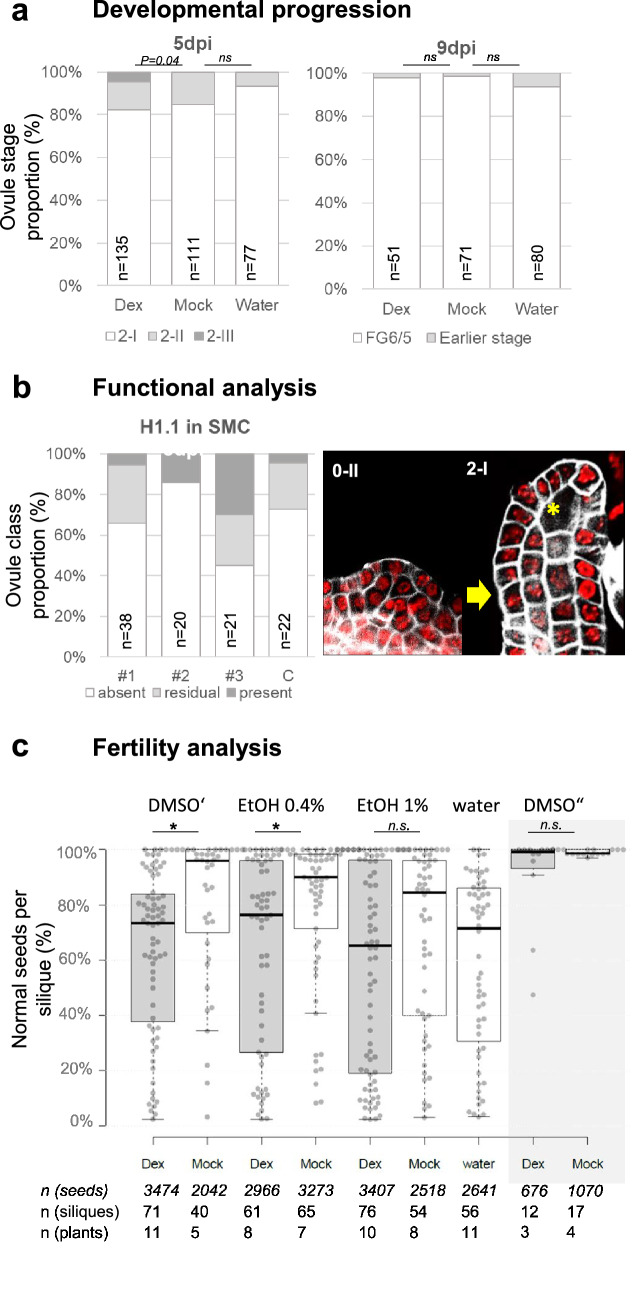
No or moderate impact of the Dex-treatment on ovule development and fertility. **a** The developmental progression of ovules following treatment was monitored by scoring ovule stages in flowers at stage 9/10 sampled at 5 dpi and flowers stage 12 sampled at 9 dpi following the nomenclature in [22, 42], n = number of ovules, P value: return from a Chi-square test (3 × 3 contingency table), *ns*: not significant. **b** The functionality of an induced construct was assessed by monitoring the eviction of H1.1-RFP during SMC differentiation as described [25]. stage 1-II ovule primordia (flower stage 9 sampled at 5 dpi) were scored for the absence, presence or residual presence of H1.1-RFP in the SMC (white, pale and dark grey, respectively); #1–3: three independent *RPS5a* >  > *H1.1-RFP* lines, C, control line (*H1.1::H1.1-RFP*). **c** Seed set per silique produced from flowers treated individually with Dex, Mock or water as indicated and using different solvents for the Dex solution (see text). The plot represents the outcome of two experiments (white and grey background, see text). Boxplot: Each dot (circle) indicates the % of normal seeds in one silique; Center lines show the medians; box limits indicate the 25th and 75th percentiles as determined by R software; the number of seeds, siliques and plants used for each experiment is indicated in the table below the plot. *P < *0.05* (Mann Whitney U test), *ns* not significant. See Additional file 1 for source data

For monitoring the developmental progression we induced flower buds at stage 5/6 and sampled floral buds at stage 9/10 (sporogenesis stage) five days post induction (5 dpi), and stage 12-buds (gametophytic stage) at 9 dpi. We cleared the ovules and scored their developmental stage according to the nomenclature [22]. If the treatment has a negative impact on development, we should observe a larger fraction of younger ovules, i.e., delayed in their developmental progression, in the Dex-treated samples compared to the Mock or water samples. Our results do not indicate such a developmental delay neither at 5 dpi or 9 dpi, (Fig. 4a; Additional file 1). Although the Dex-treated sample at 5 dpi showed a small fraction of ovules at stage 2-III not scored in the controls (generating a moderate difference (p = 0.04) in stage distribution with the Mock control), we did not interpret this as a relevant phenotype impacting developmental progression.

To address the question as whether induction affects SMC differentiation, we monitored the developmental dynamic of an inducible H1.1-RFP marker (*pRPS5a* >  > *H1.1-RFP* line). H1.1 is expressed in all cells of young ovule primordia but evicted in the SMC of stage 1-II ovule primordia. The turnover of this protein is a marker of the somatic-to-reproductive transition [25]. We induced flower buds at stage 5–6 of plants expressing a Dex-inducible H1.1-RFP variant or, as a control, a non-inducible H1.1-RFP variant expressed under its own promoter [25]. The next day (16 hpi), we collected some of the induced floral buds and found H1.1-RFP expressed throughout the primordium as expected (Fig. 4b). Five days post induction (5 dpi) we collected flower buds at stage 9/10 containing ovule primordia stage 1-II/2-I [22] and scored for the eviction process. We found that a majority of ovule primordia showed H1.1 eviction in the SMC in both the Dex-inducible and control lines (Fig. 4b; Additional file 1). Thus, the Dex induction treatment did not compromise the developmental progression of ovule primordia and the differentiation of the SMC.

Finally, to verify whether the treatment affected fertility, we treated 60 plants with a Dex, a Mock or a Water solution. Individual inflorescences were manually induced, and 423 mature siliques (18 dpi) were scored for the number of normal seeds vs infertile ovules. In this setup, 25–54% of the siliques showed a strong reduction in seed set (less than 70% seeds), including on plants subjected to treatment with a water solution (Fig. 4c; Additional file 1). Reduced fertility was essentially due to infertile ovules and not to aborted seeds, indicating a strong impact of the manual handling possibly leading occasionally to tissue injury. This was confirmed by a second experiment performed by a different user and at a moderate scale (7 plants, 27 siliques, 1746 seeds scored): the latter yielded highly fertile siliques with an average seed set of > 90% per silique without significant differences between the Mock and Dex treatment (Fig. 4c, grey panel, Additional file 1). In addition, the first (large-scale) experiment included solutions prepared with different solvents (0.25% DMSO, 0.4% EtOH, 1% EtOH). Comparing seed sets globally showed a moderate effect of the Dex itself (p < 0.01, Mann Whitney U-Test) when dissolved in DMSO and EtOH though not consistently between the EtOH dilutions (Additional file 1). The second replicate experiment, however, did not show a significant effect of the Dex (p = 0.6). We thus conclude that manual handling and the user is a major component of sterility following induction, but this can be avoided upon increased practice and in scaling experiments appropriately.

## Discussion

Chemically inducible systems offer the ability to temporally control transgene expression. In combination with tissue-specific promoters driving the activator construct, such systems ultimately allow for fine tuning gene activation at a spatial level. However, application of the inducer remains problematic when the target cells ore tissues are deeply embedded, such as meiocytes and gametes developing within the sexual organs (carpel, anthers) of the flower. In addition, induction by watering is not always an option if transgene activation is expected to be deleterious to plant development and may induce adverse effects ahead of the stage of study. To circumvent this, local application of the inducer is preferable. We designed and tested a protocol enabling robust, rapid and efficient induction throughout the carpels and anthers of an inflorescence. The application formula contains the widely used non-toxic detergent Silwet L-77 [23] at a 0.01% concentration which proved essential for a good penetration of the inducer in the floral tissue. We found that one application of 10 µM Dex with a soft-hair paintbrush to whole inflorescences is enough to allow a robust induction of transgene expression in all ovules of the carpel. We formulated a series of recommendations, partly based on empirical observations, partly quantified, and presented here. Manual handling, which we tried to describe as detailed as possible, remains an important factor influencing the induction success, linked with the integrity of the flower bud which can affect signal detection on short term or fertility on long term. When all considerations as described in this protocol are met, however, a trained user achieves 100% induction efficiency for both the number of flowers and ovules within the carpel. Beyond the examples provided here, we have induced > 1000 flowers for > 20 different constructs in the past years with 90–100% success rate.

The pOP/LhGR cassette contains a *GUS* reporter gene which can be used to test for induction efficiency before in depth-analysis of the induced construct-of-interest. This is particularly interesting when downstream analyses are demanding in terms of sample preparation. In that case for instance, each inflorescence can be controlled the next day by sampling one flower bud for GUS staining while the rest of the inflorescence is left to develop until the desired developmental stage for sample preparation and molecular or histological analyses.

Compared to other chemical induction using ethanol [1] or estradiol [2] the pOP/LhGR system has several advantages, including a tight control of gene transactivation, versatility of application among plant species and tissue/cell-types (see [8, 9] and references in the introduction). But, so far, not many studies used the pOP/LhGR system directly on reproductive organs. Dex-mediated induction either of directly GR-tagged proteins or via the pOP/LhGR system has been reported for studying developmental or cellular processes in Arabidopsis ovules and embryo [13, 26]. In these studies, Dex-treatment was usually applied in a systematical manner via watering or sprayed on the whole plant. This is a good solution when the promoter used in the driver construct is temporally and spatially very specific. However, for developmental studies covering ovule development, starting from primordium emergence until the mature female-gametophyte bearing ovule, it is challenging to find stage-specific promoters. For instance, the *KNUCKLE* gene promoter used to drive a YFP reporter specifically in the female SMC [27] is in fact also active during shoot apical meristem development [28]. This is also true for many other promoters formally used in reporter constructs for ovule development analyses. In such cases, whole-plant induction may well create adverse effects ahead of the stage of interest at which the transgene is targeted, potentially interfering with a cellular process to be studied during ovule development. Hence, the application procedure as reported here enables the use of constitutive or tissue-specific promoters yet induces transactivation only during a specific developmental window. This extends the spectrum of application of the pOP/LhGR Dex-inducible system [3, 8, 9] to young Arabidopsis ovules or anthers, notably for functional approaches. One strategy is based on conditional gene downregulation, using customised artificial microRNA or RNA interference. Notwithstanding, and like in approaches relying on stably expressed regulators, appropriate controls must be designed to measure the effectiveness of downregulation which depend both on the stability of the induced miRNA or RNAi, as well as the persistence of Dex in the tissue which is not known for ovules or anthers. Another strategy is the conditional expression of an engineered protein to decipher, for instance, the role of specific protein variants, protein domains, or residues in reproductive development. In both cases, the experimental design must carefully consider the induction vs observation time points to sample the desired developmental stage. For instance, in our growth conditions, inducing inflorescences containing flowers at stage 5/6 (ovule primordia just emerging from the placenta [22]) allowed us to follow consecutive stages by sampling five days post induction for primordia stage 1-II/2-I in flowers at stage 9/10, or nine-days post induction for mature ovules in flowers stage 12, or to assess fertility in mature siliques ~ 18 days post induction. The induction-observation timing needs to be adapted to each experiment to fit the developmental progression influenced by growth conditions and to capture the developmental process of interest: for instance ovule patterning takes several days [22, 29] while meiosis takes place within a few hours [30, 31].

## Conclusion

Arabidopsis ovules are difficult to study microscopically due to their relative inaccessibility in the carpel, and to perturb genetically due to a lack of specific promoters enabling cell- and stage-specific perturbations. The pOP/LhGR, Dex-induction system offers a good solution to induce transgenes expression locally and at a specific stage. We present here an application protocol, that provides efficient and reliable induction in the Arabidopsis ovule and in anthers, preserving the integrity of the developmental process, making this approach suitable for functional studies. In combination with the increasing number of fluorescent reporters and associated microscopy approaches used for resolving developmental processes in the reproductive organs [30–33], inducible gene transactivation, as described, here completes the experimental toolkit for ovule and anther developmental analysis.

## Methods

### Plant material and growth conditions

Arabidopsis seeds were sterilized (10 min in 0.03% bleach, 0.05% Triton X-100), washed three times in water and briefly incubated in 70% EtOH before sowing on germination medium (0.5xMurashige and Skoog (MS) salts (CAROLINE 19-5700, USA), 1% (w/v) Bactoagar pH 5.6) or storing dry on filter paper (4 °C) until sown. Seeds were stratified 2 to 4 days at 4 °C before transfer to growth incubators (Percival) with long day conditions (16 h light [120 μE m-2s-1] at 21 °C and 8 h dark at 16 °C). 2 weeks after germination, seedlings were transferred to soil and grown under long day conditions (16 h light/8 h dark; 22 °C/18 °C, respectively).

The *nls3xmVenus* construct (Joop Vermeer, University of Zurich) was subcloned in a Gateway LR reaction into the *pRPS5a-pop6-LhGR2* vector [9]. The construct was introduced into *Agrobacterium tumefaciens* (GV3101) and transformed to Arabidopsis Col-0 plants. Primary transformants were selected based on BASTA resistance, and scored as positive based on GUS reporter assays and *nls3xmVenus* signals 16 h following a 10 μM Dex-treatment.

The *H1.1-RFP* (*pRPS5a* >  > *H1.1-RFP*) construct was made by assembling a synthetic H1.1-tagRFP-T coding sequence (H1.1: AT1G06760, tagRFP-T: GenBank: ACD03281 [34], synthesis: GeneART, Invitrogen) into the *pRPS5a-pop6-LhGR* vector [9]. The constructs were transformed via *Agrobacterium tumefaciens* (GV3101) to Col-0 plants. Positive T1s were identified based on BASTA resistance selection, GUS reporter assay and RFP signals 16 h after 10 µM Dex-treatment.

The *pRPS5a* >  > *∆Kinesin-YFP* construct was made by amplifying a 957 bp fragment encompassing the coiled-coil domains of At3g20150 (recently termed Kinesin-12F [35] from Arabidopsis Col-0 cDNA using primers 5′-TTTGGCGCGCCCACCATGGGAGCAAGTAATGGAGA-3′ and 5′-AAAGGCGCGCCTTGCTCCCTACATACCTTCTTCCTC-3′. Following AscI digestion of the PCR product, the fragment was inserted into pENTRY-YFP [36]. This was then recombined with pOpIN2 [9] through an LR reaction to generate the vector *pRPS5a* >  > *∆Kinesin-YFP* (alternative name: pOpIN2/RPS5a > Dex > KIN12F-coiled-coil).

For *pSPL* >  > *amiMET1* and *pSPL* >  > *amiFIE* the putative promoter regions plus the 5’UTR and the 3’UTR of the *SPL* locus (AT4G27330) were amplified as described [37, 38]. The fragments were cloned into pBIN-LR-LhGR2 using Gateway-compatible sites as described [9] to generate the *pSPL::LhGR* vector and an AscI *pSPL::LhGR* fragment was cloned into pOPIn2 [9] to generate *pSPL::LhGR/pOPIn2*. The amiRNAs against *FIE* (AT3G20740) and *MET1* (AT5G49160) were amplified using genomic DNA Col_0 as template as described [39]. The amiRNA target sequences were TCGTCCAATTCCCTGTATTA and TAGCCAACAGAGTATTACTGC for *FIE* and *MET1*, respectively. The amiRNA were cloned into a PUC Entry clone and subcloned by LR reaction (Invitrogen) into the *pSPL::LhGR/pOPIn2* vector.

### Preparation of Dex-stock and induction solution

The stock solution of 10 mM Dex in either pure DMSO or 100% EtOH is stored at – 20 °C and can be kept for up to one year. The final induction solution consists of 10 µM Dex (SIGMA-ALDRICH, D4902) (prepared from the stock solution by diluting with water) and 0.01% Silwet L-77 (Lehle Seeds, USA, Cat. VIS-01). Importantly, the induction solution should be prepared freshly and stored at 4 °C for a maximum of three days.

### *β*-Glucuronidase reporter assay

Carpels are gently opened under the dissecting scope, immerged in GUS staining solution (Triton X-100 10%, EDTA 10 mM, Ferrocyanide 2 mM, Ferricyanide 2 mM, Na2HP04 100 mM, NaH2P04 100 mM, *β*-Glucuronidase 2 mM) and vacuum infiltrated for 5 min. The samples are then incubated for 2 h at 37 °C, briefly rinsed with 50 mM phosphate buffer (pH 6.8) (0.2 M NaH_2_PO_4_, 2M Na_2_HPO_3_) and mounted freshly in 80% glycerol for microscopy imaging (DIC settings, Leica DMR, Leica microsystems GmbH, Germany).

### Fluorescence imaging and image processing

Carpels were collected from treated flowers and placed on a clean microscope slide. Ovule primordia were gently exposed using dissecting needs in the mounting solution consisting in 0.5xMS or renaissance staining solution (4% paraformaldehyde; 1:2000 renaissance; 10% glycerol; 0.05% DMSO in 1 × PBS [modified from [40]]. Images were collected using a confocal laser scanning microscope using a 63XGLY APO NA1.4 objective (SP5R, Leica microsystems, Germany). Images were processed (partial or full projections, 3D rendering and orthogonal sections, as indicated in the figure legend) using Imaris (Bitplane, Switzerland).

### RT-PCR analysis

Quantitative real-time RT-PCR experiments were performed using cDNA obtained from inflorescences containing flower buds’ stage 6–10. Total RNA was extracted using the Qiagen RNA extraction Kit. Ambion TURBO DNA-free DNase kit was used to eliminate genomic DNA contamination according to the manufacturer's instructions (http://www.ambion.com/). The ImProm-IITM reverse transcription system (Promega) was used to retro-transcribe the treated RNA. Transcripts were detected using a Sybr Green Assay (iQ SYBR Green Supermix; Bio-Rad) using *UBIQUITIN10* (AT4G05320) as a reference gene. Assays were done in triplicate using a Bio-Rad iCycler iQ Optical System (software v.3.0a). Forward and reverse primers for FIE: TCTGAACACCTGCCTCACAG and TGTGACTGAGAACCGCTGTC, respectively; for *MET1*: GTGTGGCGTTAATGGGAAC and TCTCCATGACCCACAAGACTC, respectively. Primer specificity tests and qPCR experiments were performed as described [41] with the following cycling conditions: 3 min at 95 °C followed by 45 cycles of 10 s at 95 °C, 1 s at 55 °C, 30 s at 72 °C, and 15 s at the optimal acquisition temperature. *FIE* and *MET1* transcript levels were normalized with *UBQ10* levels.

### Scoring of ovules stages

For experiments shown in Fig. 4a, floral buds were fixed in Acetic Acid:EtOH 3:1, stored overnight at 4 °C, rinsed in 70% EtOH and mounted in clearing solution (chloral hydrate: water: glycerol 8:2:1 v:v:w) before imaging with a wide-field light microscopy (DMR, Leica microsystems, Germany). Ovule stages were scored according to the nomenclature for ovule primordia [22, 29] and ovules [42].

### Fertility analysis

Single inflorescences were induced twice (at 0 and 16 h) with 10 µM Dex, 0.01% Silwet or Mock solutions including the equivalent share of EtOH or DMSO as indicated on the graph. After 2 weeks the first 10 siliques of the induced inflorescences were collected for seed set analysis by scoring normal seeds, abnormal seeds and infertile ovules (Additional file 1).

### Data analysis and graphs

Data as presented in the Additional file 1 were plotted using Excel (Figs. 2e, 4a, b) or http://shiny.chemgrid.org/boxplotr/ (Fig. 3c; Additional file 2: Fig. S1). The two-tailed t-test Fig. 2e was computed in Excell, the Chi-square test Fig. 4a was computed using the online calculator https://www.icalcu.com/stat/chisqtest.html, the Mann–Whitney U test Fig. 4c was computed using R (Wilcoxon rank sum test, unpaired).

## Supplementary Information

**Additional file 1.** Source data for Fig. 3e and Fig. 4. Excel files containing raw and processed data (tables) shown in graphs Fig. 2 and Fig. 4

**Additional file 2: Figure S1.** Repeated induction improves the rate of success. Flower buds were induced as described in the protocol either once (one induction) or twice (two inductions, the second one on the next day). Induction success was scored following reporter gene assay (GUS or RFP, different lines were used in this study) in 9 and 10 independent experiments for one or two inductions, respectively (datapoint on the boxplot), each consisting in 3–85 flower buds (see table).

## Data Availability

The reporter lines used in the study are available from the corresponding author upon request.

## References

[1] Roslan HA, Salter MG, Wood CD, White MR, Croft KP, Robson F (2001). Characterization of the ethanol-inducible alc gene-expression system in Arabidopsis thaliana. Plant J.

[2] Zuo J, Niu QW, Chua NH (2000). Technical advance: an estrogen receptor-based transactivator XVE mediates highly inducible gene expression in transgenic plants. Plant J.

[3] Craft J, Samalova M, Baroux C, Townley H, Martinez A, Jepson I (2005). New pOp/LhG4 vectors for stringent glucocorticoid-dependent transgene expression in Arabidopsis. Plant J.

[4] Vlad D, Abu-Jamous B, Wang P, Langdale JA (2019). A modular steroid-inducible gene expression system for use in rice. BMC Plant Biol.

[5] Li XR, Deb J, Kumar SV, Ostergaard L (2018). Temperature modulates tissue-specification program to control fruit dehiscence in Brassicaceae. Mol Plant.

[6] Samalova M, Brzobohaty B, Moore I (2005). pOp6/LhGR: a stringently regulated and highly responsive dexamethasone-inducible gene expression system for tobacco. Plant J.

[7] Hajheidari M, Wang Y, Bhatia N, Vuolo F, Franco-Zorrilla JM, Karady M (2019). Autoregulation of RCO by low-affinity binding modulates cytokinin action and shapes leaf diversity. Curr Biol.

[8] Schurholz AK, Lopez-Salmeron V, Li Z, Forner J, Wenzl C, Gaillochet C (2018). A comprehensive toolkit for inducible, cell type-specific gene expression in Arabidopsis. Plant Physiol.

[9] Samalova M, Kirchhelle C, Moore I (2019). Universal methods for transgene induction using the dexamethasone-inducible transcription activation system pOp6/LhGR in Arabidopsis and other plant species. Curr Protoc Plant Biol..

[10] Moore I, Samalova M, Kurup S (2006). Transactivated and chemically inducible gene expression in plants. Plant J.

[11] Gomez MD, Barro-Trastoy D, Escoms E, Saura-Sanchez M, Sanchez I, Briones-Moreno A, et al. Gibberellins negatively modulate ovule number in plants. Development. 2018;145(13). 10.1242/dev.163865 PMC6053663 29914969

[12] Samalova M, Moore I (2021). The steroid-inducible pOp6/LhGR gene expression system is fast, sensitive and does not cause plant growth defects in rice (Oryza sativa). BMC Plant Biol.

[13] Kawashima T, Maruyama D, Shagirov M, Li J, Hamamura Y, Yelagandula R, et al. Dynamic F-actin movement is essential for fertilization in *Arabidopsis thaliana*. Elife. 2014;3. 10.7554/eLife.04501 PMC4221737 25303363

[14] Shi D, Lebovka I, Lopez-Salmeron V, Sanchez P, Greb T. Bifacial cambium stem cells generate xylem and phloem during radial plant growth. Development. 2019;146(1). 10.1242/dev.171355 PMC6340147 30626594

[15] Caggiano MP, Yu X, Bhatia N, Larsson A, Ram H, Ohno CK, et al. Cell type boundaries organize plant development. Elife. 2017;6. 10.7554/eLife.27421 PMC5617630 28895530

[16] Kannangara R, Branigan C, Liu Y, Penfield T, Rao V, Mouille G (2007). The transcription factor WIN1/SHN1 regulates Cutin biosynthesis in *Arabidopsis thaliana*. Plant Cell.

[17] Baroux C, Blanvillain R, Moore IR, Gallois P (2001). Transactivation of BARNASE under the AtLTP1 promoter affects the basal pole of the embryo and shoot development of the adult plant in Arabidopsis. Plant J.

[18] Schoof H, Lenhard M, Haecker A, Mayer KF, Jürgens G, Laux T (2000). The stem cell population of Arabidopsis shoot meristems in maintained by a regulatory loop between the CLAVATA and WUSCHEL genes. Cell.

[19] Binarova P, Cenklova V, Prochazkova J, Doskocilova A, Volc J, Vrlik M (2006). Gamma-tubulin is essential for acentrosomal microtubule nucleation and coordination of late mitotic events in Arabidopsis. Plant Cell.

[20] Yamaguchi N, Wu MF, Winter CM, Berns MC, Nole-Wilson S, Yamaguchi A (2013). A molecular framework for auxin-mediated initiation of flower primordia. Dev Cell.

[21] Pinto SC, Mendes MA, Coimbra S, Tucker MR (2019). Revisiting the female germline and its expanding toolbox. Trends Plant Sci.

[22] Schneitz K, Hülskamp M, Pruitt RE (1995). Wild-type ovule development in Arabidopsis thaliana: a light microscope study of cleared whole-mount tissue. Plant J.

[23] Clough SJ, Bent AF (1998). Floral dip: a simplified method for Agrobacterium-mediated transformation of *Arabidopsis thaliana*. Plant J.

[24] Takacs M, Ekiz-Gucer N, Reisch J, Gergely-Zobin A (1991). The light sensitivity of corticosteroids in crystalline form. Photochemical studies. Pharm Acta Helv.

[25] She W, Grimanelli D, Rutowicz K, Whitehead MW, Puzio M, Kotlinski M (2013). Chromatin reprogramming during the somatic-to-reproductive cell fate transition in plants. Development.

[26] Gross-Hardt R, Lenhard M, Laux T (2002). WUSCHEL signaling functions in interregional communication during Arabidopsis ovule development. Genes Dev.

[27] Tucker MR, Okada T, Hu Y, Scholefield A, Taylor JM, Koltunow AM (2012). Somatic small RNA pathways promote the mitotic events of megagametogenesis during female reproductive development in Arabidopsis. Development.

[28] Payne T, Johnson SD, Koltunow AM (2004). KNUCKLES (KNU) encodes a C_2_H_2_ zinc-finger protein that regulates development of basal pattern elements of the Arabidopsis gynoecium. Development.

[29] Hernandez-Lagana E, Mosca G, Sato EM, Pires N, Frey A, Giraldo-Fonseca A (2020). Organ geometry channels cell fate in the Arabidopsis ovule primordium. BioRxiv..

[30] Valuchova S, Mikulkova P, Pecinkova J, Klimova J, Krumnikl M, Bainar P, et al. Imaging plant germline differentiation within Arabidopsis flowers by light sheet microscopy. Elife. 2020;9. 10.7554/eLife.52546 PMC7012603 32041682

[31] Prusicki MA, Keizer EM, van Rosmalen RP, Komaki S, Seifert F, Muller K, et al. Live cell imaging of meiosis in *Arabidopsis thaliana*. Elife. 2019;8. 10.7554/eLife.42834 PMC6559805 31107238

[32] Mendocilla Sato E, Baroux C (2017). Analysis of 3D cellular organization of fixed plant tissues using a user-guided platform for image segmentation. Bio-Protoc.

[33] Tofanelli R, Vijayan A, Scholz S, Schneitz K (2019). Protocol for rapid clearing and staining of fixed Arabidopsis ovules for improved imaging by confocal laser scanning microscopy. Plant Methods.

[34] Shaner NC, Lin MZ, McKeown MR, Steinbach PA, Hazelwood KL, Davidson MW (2008). Improving the photostability of bright monomeric orange and red fluorescent proteins. Nat Methods.

[35] Muller S, Livanos P (2019). Plant Kinesin-12: localization heterogeneity and functional implications. Int J Mol Sci.

[36] Boehm JS, Zhao JJ, Yao J, Kim SY, Firestein R, Dunn IF (2007). Integrative genomic approaches identify IKBKE as a breast cancer oncogene. Cell.

[37] Ito T, Wellmer F, Yu H, Das P, Ito N, Alves-Ferreira M (2004). The homeotic protein AGAMOUS controls microsporogenesis by regulation of SPOROCYTELESS. Nature.

[38] Yang WC, Ye D, Xu J, Sundaresan V (1999). The SPOROCYTELESS gene of Arabidopsis is required for initiation of sporogenesis and encodes a novel nuclear protein. Genes Dev.

[39] Ossowski S, Schwab R, Weigel D (2008). Gene silencing in plants using artificial microRNAs and other small RNAs. Plant J.

[40] Musielak TJ, Schenkel L, Kolb M, Henschen A, Bayer M (2015). A simple and versatile cell wall staining protocol to study plant reproduction. Plant Reprod.

[41] Burton RA, Shirley NJ, King BJ, Harvey AJ, Fincher GB (2004). The CesA gene family of barley. Quantitative analysis of transcripts reveals two groups of co-expressed genes. Plant Physiol.

[42] Yadegari R, Drews GN (2004). Female gametophyte development. Plant Cell.

[43] Smyth DR, Bowman JL, Meyerowitz EM (1990). Early flower development in Arabidopsis. Plant Cell.

