# Supplementary material for: A procedure for Dex-induced gene transactivation in Arabidopsis ovules
Source: Plant Methods. 2022 Mar 29;18:41. doi: 10.1186/s13007-022-00879-x (PMC8962214; doi:10.1186/s13007-022-00879-x)
Supplement: Supplementary file 2 — Additional file 2: Figure S1. Repeated induction improves the rate of success. Flower buds were induced as described in the protocol either once (one induction) or twice (two inductions, the second one on the next day). Induction success was scored following reporter gene assay (GUS or RFP, different lines were used in this study) in 9 and 10 independent experiments for one or two inductions, respectively (datapoint on the boxplot), each consisting in 3–85 flower buds (see table). [file 13007_2022_879_MOESM2_ESM.docx]

**% success**

**# induction(s)**

*Table related to the graph. Each entry corresponds to an independent experiment. n, number of flower buds scored*


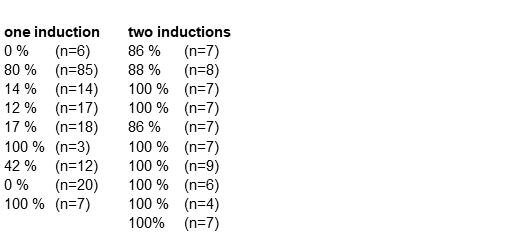


positively induced ovules: 100% (n=83)

**Figure S1. Repeated induction improves the rate of success.**

Flower buds were induced as described in the protocol either once (one induction) or twice (two inductions, the second one on the next day). Induction success was scored following reporter gene assay (GUS or RFP, different lines were used in this study) in 9 and 10 independent experiments for one or two inductions, respectively (datapoint on the boxplot), each consisting in 3-85 flower buds (see table). *Boxplot generated by: http://shiny.chemgrid.org/boxplotr/*
